# Volunteer Participation in the Health eHeart Study: A Comparison with the US Population

**DOI:** 10.1038/s41598-017-02232-y

**Published:** 2017-05-16

**Authors:** Xiaofan Guo, Eric Vittinghoff, Jeffrey E. Olgin, Gregory M. Marcus, Mark J. Pletcher

**Affiliations:** 1 Department of Cardiology, the First Hospital of China Medical University, Shenyang, Liaoning China; 2 Department of Epidemiology and Biostatistics, University of California, San Francisco, San Francisco, California USA; 3 Division of Cardiology, Department of Medicine, University of California, San Francisco, San Francisco, California USA

## Abstract

Direct volunteer “eCohort” recruitment can be an efficient way of recruiting large numbers of participants, but there is potential for volunteer bias. We compared self-selected participants in the Health eHeart Study to participants in the National Health And Nutrition Examination Survey (NHANES) 2013–14, a cross-sectional survey of the US population. Compared with the US population (represented by 5,769 NHANES participants), the 12,280 Health eHeart participants with complete survey data were more likely to be female (adjusted odds ratio (ORadj) = 3.1; 95% confidence interval (CI) 2.9–3.5); less likely to be Black, Hispanic, or Asian versus White/non-Hispanic (ORadj’s = 0.4–0.6, p < 0.01); more likely to be college-educated (ORadj = 15.8 (13–19) versus ≤high school); more likely to have cardiovascular diseases and risk factors (ORadj’s = 1.1–2.8, p < 0.05) except diabetes (ORadj = 0.8 (0.7–0.9); more likely to be in excellent general health (ORadj = 0.6 (0.5–0.8) for “Good” versus “Excellent”); and less likely to be current smokers (ORadj = 0.3 (0.3–0.4)). While most self-selection patterns held for Health eHeart users of Bluetooth blood pressure cuff technology, there were some striking differences; for example, the gender ratio was reversed (ORadj = 0.6 (0.4–0.7) for female gender). Volunteer participation in this cardiovascular health-focused eCohort was not uniform among US adults nor for different components of the study.

## Introduction

Emerging technology, near-ubiquitous access to the internet, and ease of electronic communication makes it possible to contact and recruit participants over the internet, consent and collect data without in-person visits, and repurpose new sensor devices and smartphone technology for longitudinal research data collection. This so-called “eCohort” approach can be an extremely efficient epidemiologic approach that is attractive in an era of shrinking funds for traditional studies^1^. Even the well-endowed Precision Medicine Initiative will employ internet- and mobile phone application- (app-) based recruitment to recruit over a third of the planned 1 million person cohort^2^.

This approach, however, may yield substantial volunteer bias. Technology use is not uniform in the US^3^, and reliance on response to electronically-delivered invitations for study participation is likely to select for particular individuals with favorable impressions of the research establishment, strong altruistic motivation, and time to complete research activities. Prior internet-based surveys, for example, have reported over-representation of women, married, and well-educated individuals^4^. No prior analyses have reported on internet-based recruitment into a US-based eCohort in comparison with the US population.

The Health eHeart Study is a large eCohort study focused on cardiovascular health. Health eHeart invites any adult age 18 years or older with an email address to participate, recruits primarily over the internet via electronically-delivered invitations, collects surveys and patient-reported outcomes, and supports connection of a wide variety of consumer electronic devices and apps to the study so that the mHealth data they collect can be donated and delivered to the Health eHeart Study database. We compared participants in the Health eHeart Study to participants in the National Health And Nutrition Examination Survey (NHANES), which was designed to be representative of the US population, for the purpose of informing inferences made using Health eHeart Study analyses and for targeting recruitment to balance our study sample.

## Methods

### Health eHeart Study Sample

The Health eHeart Study is a cardiovascular focused eCohort, with enrollment, consent and participant occurring entirely using the internet. We analyzed cross-sectional baseline examination data and follow-up data from Bluetooth-enabled blood pressure measurement devices obtained between March 8, 2013 (enrollment initiation) and March 24, 2016 from consecutive participants enrolled in the Health eHeart Study. Participation in the Health eHeart Study is open to any person (world-wide) with a self-reported date of birth indicating age ≥18 years and an email address. Recruitment into the study occurred via several news media stories, social media and word-of-mouth in addition to being actively sought via email campaigns sent to persons associated with the American Heart Association (primarily via emails sent to participants in their Go Red for Women campaign^5^), to adult patients at the University of California, San Francisco (UCSF) Medical Center (primarily via unsolicited email invitation), through various other specific referral sources (we track referral source by provided a special URL to referring partners), and from unspecified sources (through our general URL).

After online registration (name, date of birth, email and password) and consent, participants were prompted to complete a series of online questionnaires pertaining to basic socio-demographics, family history, medical history, activity and well-being, habits and lifestyle, mental health, food and nutrition, and use of internet or social media. Participants were also invited to “connect” devices and apps (that they already own) from Fitbit, iHealth, Withings, Qardio, Alivecor, Azumio, Ginger.io and Google Fit and donate their data to the study. We limited our primary analysis to participants age ≥20 years (for comparability with NHANES) and with complete information and without “unknown” or “refused” responses on all baseline core survey instruments and survey items. For our secondary analysis, we additionally limited the sample to such participants who also contributed at least one blood pressure measurement via Bluetooth-enabled blood pressure measurement devices (iHealth, Withings and Qardio were all supported).

### NHANES Sample

We used NHANES 2013–2014 to represent the US population and compare against participants in the Health eHeart Study. NHANES is a program of the National Center for Health Statistics (NCHS) that aims to investigate the health and nutritional status of the US population. Since 1999, the survey has been released every 2 years in a continuous fashion. These cross-sectional data are representative of the non-institutionalized US population. Every year, approximately 5,000 individuals of all ages are interviewed in their homes and complete the health examination component of the survey. NHANES follows a complex, multistage sampling procedure where the primary sampling units are counties or small groups of contiguous counties, within which city blocks are selected. Within these blocks, households are then randomly selected, and then individuals are drawn at random^6^. All NHANES protocols were approved by the NCHS Research Ethics Review Board^7^. In 2013–2014, 14,332 persons were selected for NHANES from 30 different study locations. Of those selected, 10,175 completed the interview. NHANES provides study weights that account for both non-response and deliberate oversampling of particular segments of the population.

Because various components of NHANES are only delivered to adults ≥20 years, we limited our analyses to these participants, leading to a sample size of 5,769. In order to maintain strict representativeness of the NHANES study sample ≥20 years and allow for direct comparisons with Health eHeart, we performed multiple imputation using chained equations to estimate missing and “unknown”/non-response values of all variables of interest (n = 13 variables) for all participants (n = 1,162 participants with at least one missing value)^8, 9^. We used 10-fold multiple imputation to generate imputed datasets, each with complete data on all 5,769 NHANES participants included in our sample. This 10-fold imputed dataset was used for all subsequent analyses.

Informed consent was obtained from all participants in both Health eHeart and NHANES. Our analysis of the Health eHeart Study data is covered by the UCSF Institutional Review Board (IRB); our analysis of the de-identified NHANES data is exempt from IRB Review. Methods were performed in accordance with the relevant guidelines and regulations.

### Statistical Method

We first used descriptive statistics to compare the demographic characteristics, medical conditions, and lifestyle factors of the Health eHeart sample by recruitment source, using ANOVA and chi-square tests for between-source differences. Then, to identify factors independently associated with participation in Health eHeart, we used a case-control approach, using pooled data for the combined NHANES and Health eHeart samples to estimate logistic regression models for the “outcome” of inclusion in the Health eHeart Study sample. We first fit single-predictor models for age, sex, race, income, marriage status, educational level, hypertension, hyperlipidemia, diabetes, stroke, coronary heart disease, heart failure, heart attack, general health, smoking and sleeping duration, and then fit a final multivariable model for Health eHeart participation that included this entire set of predictors. Results are summarized as odd ratios (ORs) and 95% confidence intervals (CIs). We accounted for the complex stratified survey design of NHANES using the sampling weights, pseudo-strata, and primary sampling unit (PSU) variables provided by NHANES, with weights normalized to sum to the NHANES sample size. In the pooled analyses, Health eHeart participants were each given unit weight, and randomly assigned to two PSUs with a distinct pseudo-stratum. Multiple imputation of the NHANES data was implemented using the *mi* package in Stata Version 14.0, and the case-control models were estimated using the Stata *svy* package for complex survey data, which accommodates multiply-imputed data. Two-sided *P* values less than 0.05 were considered to be statistically significant.

## Results

**Figure 1:**
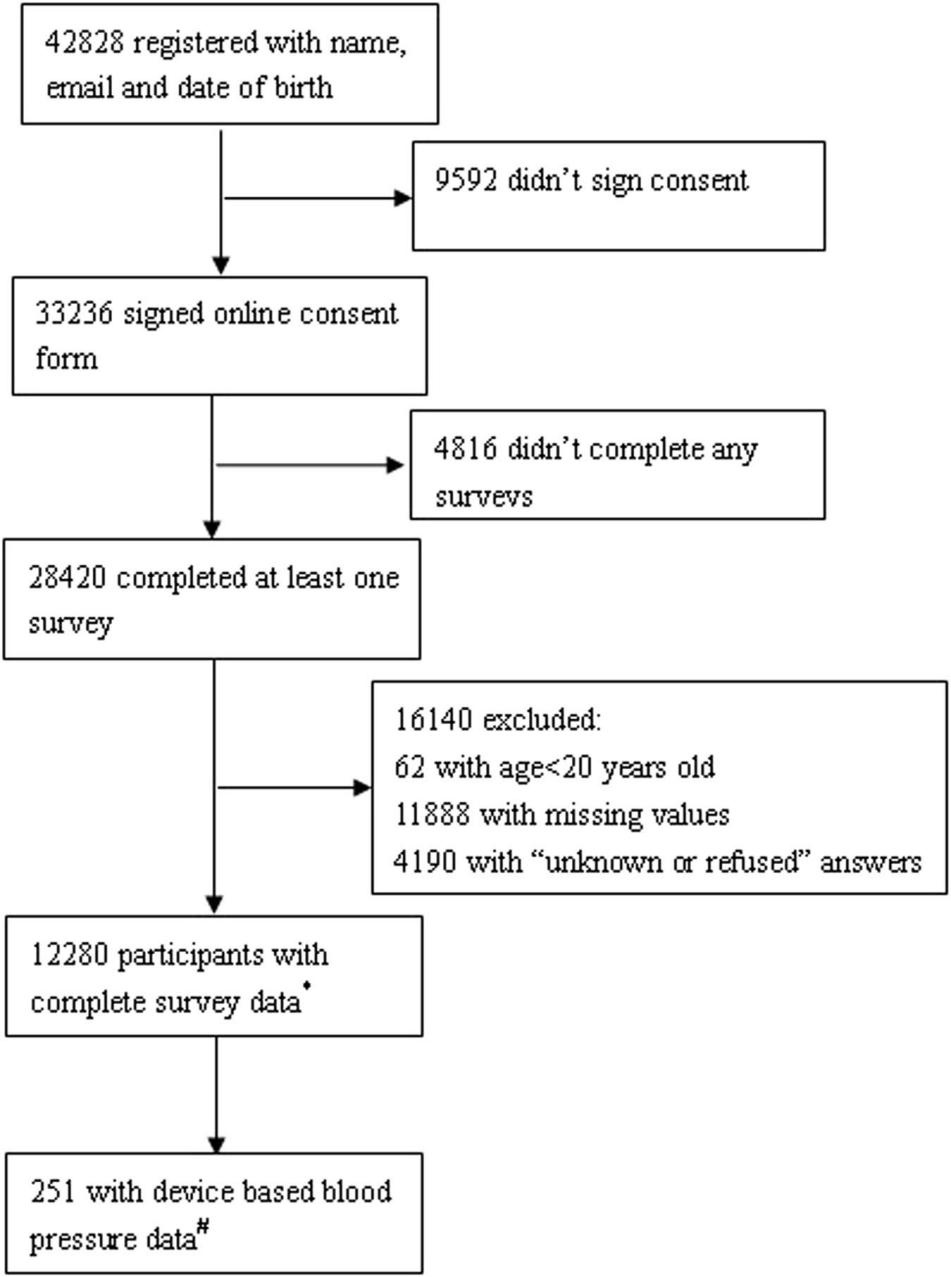
Flowchart of self-selection process in Health eHeart Study as of 24/03/2016. ^*^Health eHeart Study sample used in Tables 1 and 2. ^#^Health eHeart Study sample subset used in Table 3.

At the time of our data lock, 42,828 participants had registered for the Health eHeart Study by providing their name, email and date of birth. Of those, 33,236 (78% of registered participants) signed the online consent, 28,420 completed at least one survey, (86% of consented participants), and 12,280 were participants age ≥20 years with complete core baseline survey data and without “unknown” or “refused” responses to any survey item (Fig. 1). These participants constitute our primary analysis sample. Of these, 251 contributed at least one blood pressure measurement via Bluetooth-enabled blood pressure measurement device; these participants constitute our secondary analysis sample (Fig. 1). As described in our Methods, all NHANES participants age ≥20 years were included after multiple imputation successfully imputed missing/unknown/refused items for the 1,162 participants missing at least one required data element.

Baseline characteristics of Health eHeart Study participants differed by referral source (Table 1). For example, only 3% of participants referred by American Heart Association sources were male (consistent with the primary focus on the Go Red for Women program), compared with 37%-44% from other sources (p < 0.001). We also detected differences by recruitment source in age (more elderly participants from UCSF), race/ethnicity (more Black, non-Hispanic participants from AHA), income and education (higher in both from UCSF), general health (highest among participants from unspecified referral source), and sleep duration (lowest duration from AHA referrals, Table 1, all p-values < 0.001).

**Table 1:** Baseline characteristics of Health eHeart Study population with complete survey variables (N = 12280).

Characteristics	Referred from AHA (n = 4586)	UCSF patients (n = 2602)	Other specific referral source (n = 946)	Non-specific source (n = 4146)	*P*-value
**Age, mean years +/− SD**	50 +/− 12	57 +/− 15	52 +/− 14	50 +/− 15	<0.001
**Age group, %**					<0.001
20–29	7%	5%	7%	12%	
30–39	14%	11%	15%	16%	
40–49	24%	15%	20%	18%	
50–59	32%	21%	27%	22%	
60–69	19%	29%	21%	22%	
70–79	3%	17%	9%	9%	
≥80	0%	3%	1%	1%	
**Sex, % male**	3%	39%	37%	44%	<0.001
**Race/ethnicity**					<0.001
Black, non-Hispanic	8%	1%	2%	3%	
White, non-Hispanic	82%	83%	88%	81%	
Asian, non-Hispanic	2%	6%	3%	7%	
Hispanic	5%	7%	4%	5%	
Others or mixed	4%	4%	3%	4%	
**% Married or partnered**	68%	70%	71%	71%	0.027
**Education**					<0.001
≤High school	7%	3%	7%	4%	
Some college or associate degree	31%	15%	23%	16%	
College graduate or above	62%	82%	70%	80%	
**Annual Incom, %**					<0.001
<$20,000	9%	6%	5%	6%	
$20,000−<$75,000	43%	23%	32%	28%	
$75,000−<$100,000	17%	12%	16%	14%	
≥$100,000	32%	59%	47%	52%	
**Medical conditions, %**					
Hypertension	40%	36%	37%	33%	<0.001
Hyperlipidemia	45%	42%	44%	41%	0.001
Diabetes	10%	8%	6%	6%	<0.001
Coronary heart disease	13%	8%	10%	10%	<0.001
Stroke	4%	4%	5%	3%	0.106
Heart failure	5%	2%	7%	3%	<0.001
Heart attack	9%	3%	5%	6%	<0.001
**General health, %**					<0.001
Excellent	12%	19%	15%	23%	
Very good	33%	37%	36%	39%	
Good	38%	30%	37%	27%	
Fair	14%	11%	10%	9%	
Poor	3%	2%	3%	1%	
**Smoking, %**					<0.001
Never	66%	62%	59%	67%	
Past	29%	35%	36%	30%	
Current	5%	3%	5%	4%	
**Sleep duration, h/night**					<0.001
≤6	43%	30%	35%	31%	
7 to 8	53%	63%	60%	64%	
≥9	5%	7%	5%	5%	

Data are presented as means +/− standard deviation or percentages.

NHANES: National Health And Nutrition Examination Survey; AHA: American Heart Association; UCSF: University of California, San Francisco.

Compared with all adults in the US, as represented by NHANES participants (applying sample weights), Health eHeart Study participants were more likely to be middle-aged: more likely to be female; less likely to be Black, Hispanic, or Asian versus White/non-Hispanic; more likely to be highly educated; more likely to have cardiovascular disease and risk factors but less likely to have diabetes; more likely to be in excellent general health; less likely to be current smokers; and more likely to report low sleep duration (Table 2). Associations with higher income and marital status did not persist in adjusted models. The higher prevalence of female participants in Health eHeart persisted even after excluding participants referred from the Go Red for Women program (OR_adj_ = 1.6; 95% CI: 1.5–1.7). When we limited both the Health eHeart Study and NHANES population to participants with coronary heart disease (Health eHeart Study n = 1297; NHANES n = 293), characteristics of the sample were different (e.g., higher prevalence of cardiovascular risk factors), but predictors of participation in the Health eHeart Study were quite similar (Supplemental Table 1).

**Table 2:** Predictors of volunteering for the Health eHeart Study with reference to the National Health and Nutrition Examination Survey.

Characteristics	NHANES (N = 5769)	Heh (N = 12280)		Unadjusted	Adjusted		
		n	%	ORs (95% CIs)	*P*-value	ORs (95% CIs)	*P*-value
**Age group**							
20–29	19%	1034	8%	1 (ref)		1 (ref)	
30–39	17%	1730	14%	1.8 (1.5–2.1)	<0.001	1.4 (1.2–1.7)	0.001
40–49	19%	2392	19%	2.3 (1.9–2.8)	<0.001	1.9 (1.4–2.4)	<0.001
50–59	18%	3195	26%	3.2 (2.7–3.7)	<0.001	2.3 (1.9–2.8)	<0.001
60–69	14%	2734	22%	3.4 (2.8–4.2)	<0.001	2.2 (1.7–2.8)	<0.001
70–79	8%	1048	9%	2.4 (2.0–2.8)	<0.001	1.5 (1.2–1.9)	0.002
≥80	4%	147	1%	0.6 (0.5–0.7)	<0.001	0.3 (0.2–0.4)	<0.001
**Sex**							
Male	48%	3311	27%	1 (ref)		1 (ref)	
Female	52%	8969	73%	2.5 (2.3–2.7)	<0.001	3.1 (2.9–3.5)	<0.001
**Race/ethnicity**							
White, non-Hispanic	66%	529	82%	1 (ref)		1 (ref)	
Black, non-Hispanic	11%	10069	4%	0.3 (0.2–0.5)	<0.001	0.4 (0.3–0.5)	<0.001
Asian, non-Hispanic	5%	547	4%	0.7 (0.5–0.9)	0.018	0.6 (0.4–0.8)	0.007
Hispanic	15%	656	5%	0.3 (0.2–0.5)	<0.001	0.5 (0.3–0.8)	0.004
Others or mixed	3%	479	4%	1.1 (0.8–1.7)	0.47	1.4 (1.1–1.9)	0.024
**Married or partnered**	62%	8532	69%	1.4 (1.2–1.6)	<0.001	0.9 (0.8–1.1)	0.226
**Education**							
≤High school	37%	629	5%	1 (ref)		1 (ref)	
Some college or associate degree	33%	2687	22%	4.9 (4.0–5.9)	<0.001	4.0 (3.3–4.8)	<0.001
College graduate or above	30%	8964	73%	17.6 (13.5–22.8)	<0.001	15.8 (13.0–19.0)	<0.001
**Annual Income**, **%**							
<$20,000	15%	825	7%	1 (ref)		1 (ref)	
$20,000−<$75,000	48%	4041	33%	1.6 (1.2–2.0)	0.001	1.0 (0.7–1.3)	0.829
$75,000−<$100,000	11%	1840	15%	3.2 (2.2–4.5)	<0.001	1.3 (0.9–2.0)	0.198
≥$100,000	26%	5574	45%	3.9 (2.6–5.8)	<0.001	1.1 (0.7–1.8)	0.649
**Medical conditions**, **%**							
Hypertension	35%	4472	36%	1.1 (1.0–1.2)	0.211	1.1 (1.0–1.3)	0.073
Hyperlipidemia	35%	5288	43%	1.4 (1.3–1.5)	<0.001	1.1 (1.0–1.3)	0.029
Diabetes	10%	967	8%	0.8 (0.7–0.9)	0.001	0.8 (0.7–0.9)	0.007
Coronary heart disease	5%	1297	11%	2.5 (2.0–3.1)	<0.001	2.8 (2.0–3.8)	<0.001
Stroke	3%	466	4%	1.3 (1.1–1.6)	0.011	1.5 (1.1–2.0)	0.014
Heart failure	3%	487	4%	1.5 (1.2–1.9)	0.001	1.6 (1.3–2.1)	0.001
Heart attack	3%	779	6%	1.9 (1.6–2.4)	<0.001	1.2 (1.0–1.6)	0.059
**General health, %**							
Excellent	10%	2134	17%	1 (ref)		1 (ref)	
Very good	30%	4451	36%	0.7 (0.6–0.8)	<0.001	0.7 (0.5–0.8)	0.001
Good	40%	3984	32%	0.4 (0.4–0.5)	<0.001	0.6 (0.5–0.8)	<0.001
Fair	17%	1430	12%	0.4 (0.3–0.5)	<0.001	0.7 (0.6–0.8)	0.001
Poor	3%	281	2%	0.5 (0.3–0.6)	<0.001	0.8 (0.5–1.4)	0.488
**Smoking, %**							
Never	56%	7953	65%	1 (ref)		1 (ref)	
Past	24%	3791	31%	1.1 (1.0–1.3)	0.063	1.3 (1.1–1.5)	0.015
Current	20%	536	4%	0.2 (0.2–0.2)	<0.001	0.3 (0.3–0.4)	<0.001
**Sleep duration, h/night**							
≥9	8%	657	5%	1 (ref)		1 (ref)	
7 to 8	57%	7249	59%	1.6 (1.2–2.1)	0.004	1.4 (1.0–1.8)	0.025
≤6	35%	4374	36%	1.5 (1.1–2.2)	0.019	1.8 (1.3–2.6)	0.002

NHANES: National Health And Nutrition Examination Survey; Heh: Health eHeart Study; OR: odds ratio; 95% CI: 95% confidence interval.

Only a small subset of Health eHeart Study participants (n = 251, 2%) used a Bluetooth-enabled blood pressure measurement device, connected their device account to their Health eHeart Study account, and donated at least one blood pressure measurement to the study (median number of measurements per participant = 30; interquartile range 9–82). These highly self-selected participants showed mostly similar patterns of characteristics when compared with NHANES as the full Health eHeart sample, with some striking contrasts (Table 3). Instead of a large female preponderance in the full Health eHeart sample (73%, Table 2), Health eHeart participants contributing device-measured blood pressure values were less likely to be female than the US population (35%, Table 3). Persons with hypertension and coronary heart disease were even more heavily over-represented in this subset. Also, in this subsample in which moderately expensive purchases were required (blood pressure cuff and smartphone), higher income persisted as a strong predictor even after adjustment for education and other factors.

**Table 3:** Predictors of being in Health eHeart Study using sample with app based blood pressure against National Health and Nutrition Examination Survey.

Characteristics	NHANES (N = 5769)	Heh (N = 251)		Unadjusted	Adjusted		
		n	%	ORs (95% CIs)	*P*-value	ORs (95% CIs)	*P*-value
**Age group**							
18–29	19%	11	4%	1 (ref)		1 (ref)	
30–39	17%	32	13%	3.1 (1.0–9.7)	0.049	2.2 (0.6–7.4)	0.206
40–49	19%	59	24%	5.4 (3.5–8.5)	<0.001	3.5 (1.9–6.6)	0.001
50–59	18%	81	32%	7.5 (4.0–14.1)	<0.001	4.2 (1.9–9.4)	0.002
60–69	14%	50	20%	5.9 (2.3–15.2)	0.001	3.2 (1.2–8.5)	0.026
70–79	8%	18	7%	3.9 (1.7–9.0)	0.004	2.0 (0.9–4.7)	0.087
≥80	4%	0	0%	NA		NA	
**Sex**							
Male	48%	164	65%	1 (ref)		1 (ref)	
Female	52%	87	35%	0.5 (0.4–0.6)	<0.001	0.6 (0.4–0.7)	<0.001
**Race/ethnicity**							
White, non-Hispanic	66%	10	83%	1 (ref)		1 (ref)	
Black, non-Hispanic	11%	209	4%	0.3 (0.1–0.7)	0.007	0.5 (0.2–1.0)	0.058
Asian, non-Hispanic	5%	7	3%	0.4 (0.1–1.2)	0.089	0.4 (0.1–0.9)	0.032
Hispanic	15%	16	6%	0.3 (0.2–0.6)	<0.001	0.8 (0.5–1.4)	0.423
Others or mixed	3%	9	4%	1.0 (0.7–1.6)	0.872	1.7 (1.0–2.9)	0.05
**Married or partnered**	62%	191	76%	1.9 (1.2–3.1)	0.007	0.8 (0.5–1.3)	0.325
**Education**							
≤High school	37%	15	6%	1 (ref)		1 (ref)	
Some college or associate degree	33%	45	18%	3.4 (1.4–8.2)	0.009	2.8 (1.2–6.4)	0.017
College graduate or above	30%	191	76%	15.7 (8.1–30.4)	<0.001	8.7 (5.1–14.9)	<0.001
**Annual Income**, **%**							
<$20,000	15%	7	3%	1 (ref)		1 (ref)	
$20,000−<$75,000	48%	59	24%	2.7 (1.3–5.4)	0.009	1.9 (1.1–3.2)	0.02
$75,000−<$100,000	11%	29	12%	5.9 (2.1–16.5)	0.002	2.6 (1.3–5.4)	0.012
≥$100,000	26%	156	62%	12.8 (4.8–34.0)	<0.001	3.9 (1.9–8.1)	0.001
**Medical conditions**, **%**							
Hypertension	35%	111	44%	1.5 (1.0–2.3)	0.071	1.9 (1.2–3.2)	0.016
Hyperlipidemia	35%	108	43%	1.4 (1.3–1.5)	<0.001	0.9 (0.7–1.1)	0.373
Diabetes	10%	19	8%	0.7 (0.4–1.5)	0.383	0.8 (0.3–2.0)	0.564
Coronary heart disease	5%	22	9%	2.0 (1.4–3.0)	0.002	3.3 (2.1–5.3)	<0.001
Stroke	3%	7	3%	1.0 (0.4–2.3)	0.937	1.2 (0.8–1.9)	0.385
Heart failure	3%	7	3%	1.1 (0.5–2.5)	0.87	1.8 (0.6–5.3)	0.268
Heart attack	3%	7	3%	0.8 (0.3–2.0)	0.641	0.5 (0.2–1.1)	0.092
**General health, %**							
Excellent	10%	53	21%	1 (ref)		1 (ref)	
Very good	30%	89	35%	0.5 (0.3–1.0)	0.041	0.6 (0.3–1.0)	0.06
Good	40%	83	33%	0.4 (0.3–0.4)	<0.001	0.5 (0.4–0.7)	<0.001
Fair	17%	21	8%	0.2 (0.2–0.3)	<0.001	0.5 (0.3–0.8)	0.005
Poor	3%	5	2%	0.3 (0.1–0.8)	0.012	0.7 (0.2–2.4)	0.564
**Smoking%**							
Never	56%	149	59%	1 (ref)		1 (ref)	
Past	24%	89	35%	1.4 (1.2–1.6)	<0.001	1.5 (1.1–1.9)	0.011
Current	20%	13	5%	0.2 (0.1–0.4)	<0.001	0.5 (0.2–1.2)	0.1
**Sleep duration, h/night**							
≥9	8%	9	4%	1 (ref)		1 (ref)	
7 to 8	57%	161	64%	2.6 (2.2–2.9)	<0.001	2.4 (1.5–3.8)	0.001
≤6	35%	81	32%	2.1 (1.5–2.9)	<0.001	2.5 (1.8–3.5)	<0.001

NHANES: National Health And Nutrition Examination Survey; Heh: Health eHeart Study; OR: odds ratio; 95% CI: 95% confidence interval.

A total of 5668 participants in adjusted regression and unadjusted regression for agegroup due to zero count of participant over 80 year old in Heh.

## Discussion

The Health eHeart Study used efficient electronic methods for recruitment and took advantage of partner organizations willing to refer patients to our study website. This resulted in extremely efficient recruitment into the study. The sample of recruited individuals, however, differs from the US population in a variety of ways. Not only does the study over-represent persons with cardiovascular diseases and risk factors (as expected based on the study focus), but it also appears to over-represent females and non-Hispanic Whites, higher educational level, persons with more prevalent medical conditions but better self-reported general health, and fewer current smokers than would be expected if participation were proportional from all segments of the US population. Patterns were different (e.g., reversal of the female predominance) in the highly selected subset of the Health eHeart Study who contributed blood pressure measurements from a Bluetooth-enabled device.

Internet- and technology-enabled epidemiology can have major advantages in terms of efficiency. Consistent with the Health eHeart Study recruitment experience, one Danish internet-based study estimated more than 50% savings in their recruitment compared with a conventional approach ($160 vs. $322 per subject)^10^, and an internet-based clinical trial similarly reported that their web-based methods cost about half that of a hospital based approach^11^. Web-based questionnaires generally reduce cost substantially^12^, as do studies that invite participation by e-mail^13^. Aside from cost, web-based surveys can be more efficient in terms of response speed from respondents^14^, easier to adjust and modify by the research team^15^, quicker and less error-prone to process since data are entered electronically and coded automatically^16^, and easier to complete for disabled participants^17^.

Our results, in terms of which characteristics predicted participation, were similar in some ways, but different in others when compared with prior studies. As with Health eHeart, women and those with higher socioeconomic status appear to be consistently more likely to participate in epidemiologic studies^18^, especially in eCohorts^14, 19, 20^. For example, the NutriNet-Santé study in France found a much higher percentage of women compared with the corresponding national figures (78.0% vs 52.4%); and both the NutriNet-Santé study and the Australian Longitudinal Study on Women’s Health found higher participation rates in persons with higher educational levels. In contrast to the NutriNet-Santé study, however, which found higher proportions of married or partnered participants compared to their national data (70.8% vs. 62.0%), the unadjusted association we found in Health eHeart (69% married vs. 62% in NHANES) was not significant after adjusting for other selection factors. Also in contrast with Health eHeart, the Australian Longitudinal Study reported a *higher* percentage of study participants who rated their health in the online survey as fair or poor, and a *higher* percentage of study participants who were current smokers compared to their Census data. Their study, however, was limited to a very narrow demographic band (women age 18–23) so may not be comparable. We did not find another study describing self-selected participation in a study requiring use of sensor technology such as our analysis of participants in the Bluetooth-connected blood pressure cuff subsample.

Several factors likely contribute to the differences we observed between the Health eHeart Study and NHANES. First of all, NHANES makes special efforts to recruit underrepresented minorities. In fact, such individuals are oversampled in NHANES (though sample weights correct this factor so results are generalizable to the US population). No such efforts are made in the Health eHeart Study. Second, the Health eHeart Study’s focus naturally attracts participants at risk for heart disease, so the overrepresentation of people with cardiovascular diseases, such as coronary heart disease, stroke and heart failure, is to be expected. However, when we subset both samples to only participants with coronary heart disease, general selection patterns (e.g., for sex, race/ethnicity, education level and smoking) were consistent with those we found in the full Health eHeart sample. Clearly, the “digital divide” may explain differences in participation by education, and particularly also by income for the subset of Health eHeart using a Bluetooth-enabled blood pressure measurement device. As the digital divide diminishes^21^ and technology diffuses through all segments of society, this participation selection factor may ameliorate to some degree.

The Health eHeart Study is large and nationally-scoped and includes participants who complete extensive online surveys and device-associated data collection; and the NHANES study provides a near-ideal way to compare to the US population. However, our analysis has some limitations. Unlike NHANES, the Health eHeart Study does not limit participation to US residents. In contrast to Health eHeart, bias from self-selected non-participation in NHANES is minimized by post-stratification re-weighting based on the known demographic characteristics of the target sample; however, missing values arising from so-called item non-response in NHANES may not be missing at random (even conditional on other factors included in our imputation model), such that multiple imputation may be flawed. Finally, while both Health eHeart and NHANES collect many additional measurements, we were only able to evaluate measurements that were identically collected in both studies (or nearly so), preventing us from assessing the representativeness of Health eHeart on other potentially important dimensions.

Our results have some clear implications. First, given that Health eHeart recruitment is ongoing, this analysis provides guidance for how the study team can refocus recruitment efforts to target thus-far under-represented subgroups of the US population. It also represents a roadmap for prospective targeting efforts that can be used by the Precision Medicine Initiative as it begins internet-based direct volunteer recruitment later this year. While some self-selection characteristics may be expected from prior work on participation in research (e.g., under-representation of racial/ethnic minorities^22^), our findings regarding the technology product-dependent subsample (e.g., reversal of the sex ratio) are more surprising and potentially important to account for.

The other clear implication relates to inference: it is clear that simple descriptive analyses of the self-selected Health eHeart Study (e.g., % technology use) will often not yield results that are representative of the US population, either on average or within strata defined by other covariates (e.g., gender). However, it is important to note that estimates of average adjusted *associations* are likely robust to over- or under- (mis-) sampling even on the variables included in the association, provided that the mis-sampling occurs independently for each variable, and that the association is not modified by factors associated with self-selection. For example, we might obtain valid adjusted estimates of the marginal association of technology use with gender, despite oversampling of technology users and of women in the Health eHeart Study, provided that the oversampling on each factor is independent, and that the effect of technology use on gender does not vary, for example, by education. Note, even in the presence of effect modification, estimates within strata of the effect modifier should remain valid (e.g., there is internal validity). Furthermore, the effects of these various aspects of selection bias may potentially be minimized by re-weighting the Health eHeart sample (similar to the post-stratification weighting performed by NHANES), based on an extension of the multivariable logistic model developed here, with the result that all included covariates have weighted distributions very close to those in NHANES.

In conclusion, the Health eHeart Study demonstrates efficient internet-based recruitment, and allows remote data collection from online surveys and sensor/device technology. While it also clearly demonstrates that participants who volunteer for the study are different on average than the US population, this does not rule out its potential for providing valid estimates of adjusted associations. Whether this limitation can be overcome by future internet-based studies such as the planned Precision Medicine Initiative Cohort remains to be seen and will likely require more deliberate sampling, more costly targeted recruitment efforts, and application of post-recruitment standardization methods that correct for unrepresentative volunteer participation.

## Electronic supplementary material

Supplemental Table 1
