# Supplementary material for: Volunteer Participation in the Health eHeart Study: A Comparison with the US Population
Source: Sci Rep. 2017 May 16;7:1956. doi: 10.1038/s41598-017-02232-y (PMC5434039; doi:10.1038/s41598-017-02232-y)
Supplement: Supplementary file 1 — Supplemental Table 1 [file 41598_2017_2232_MOESM1_ESM.doc]

Supplemental Material

Volunteer Participation in the Health eHeart Study

A Comparison with the US Population

Xiaofan Guo; Eric Vittinghoff; Jeffrey E. Olgin; Gregory M. Marcus; Mark J. Pletcher

| Supplemental Table 1 Predictors of being in Health eHeart Study against National Health and Nutrition Examination Survey among participants with coronary heart disease. | | | | | | | |
| --- | --- | --- | --- | --- | --- | --- | --- |
| Characteristics | NHANES (N=293) | Heh (N=1297) | | Unadjusted | | Adjusted | |
|  |  | n | % | ORs (95% CIs) | *P*-value | ORs (95% CIs) | *P*-value |
| **Age group** |  |  |  |  |  |  |  |
| 20-39 | 5% | 64 | 5% | 1 (ref) |  | 1 (ref) |  |
| 40-49 | 6% | 184 | 14% | 2.4 (1.2-4.8) | 0.017 | 1.9 (0.6-6.4) | 0.262 |
| 50-59 | 14% | 383 | 30% | 2.2 (0.8-5.8) | 0.1 | 1.9 (0.6-6.0) | 0.242 |
| 60-69 | 29% | 411 | 32% | 1.2 (0.6-2.2) | 0.611 | 0.7 (0.2-2.7) | 0.602 |
| 70-79 | 23% | 217 | 17% | 0.8 (0.3-1.8) | 0.527 | 0.4 (0.1-1.6) | 0.192 |
| ≥80 | 21% | 38 | 3% | 0.1 (0.1-0.4) | 0.001 | 0.1 (0.0-0.3) | 0.003 |
| **Sex** |  |  |  |  |  |  |  |
| Male | 57% | 494 | 38% | 1 (ref) |  | 1 (ref) |  |
| Female | 43% | 803 | 62% | 2.2 (1.4-3.4) | 0.002 | 3.9 (2.1-7.5) | <0.001 |
| **Race/ethnicity** |  |  |  |  |  |  |  |
| White, non-Hispanic | 80% | 1131 | 87% | 1 (ref) |  | 1 (ref) |  |
| Black, non-Hispanic | 8% | 37 | 3% | 0.3 (0.2-0.6) | 0.001 | 0.3 (0.1-0.5) | 0.002 |
| Asian, non-Hispanic | 3% | 32 | 2% | 0.8 (0.5-1.4) | 0.428 | 0.7 (0.3-2.0) | 0.523 |
| Hispanic | 8% | 49 | 4% | 0.4 (0.2-0.9) | 0.035 | 0.3 (0.1-0.8) | 0.02 |
| Others or mixed | 1% | 48 | 4% | 2.3 (0.6-8.3) | 0.187 | 1.8 (0.5-6.3) | 0.34 |
| **Married or partnered** | 61% | 940 | 72% | 1.7 (1.3-2.0) | <0.001 | 0.8 (0.6-1.2) | 0.294 |
| **Education** |  |  |  |  |  |  |  |
| ≤ High school | 46% | 119 | 9% | 1 (ref) |  | 1 (ref) |  |
| Some college or associate degree | 30% | 405 | 31% | 5.2 (3.5-7.9) | <0.001 | 4.6 (2.8-7.7) | <0.001 |
| College graduate or above | 24% | 773 | 60% | 12.4 (7.3-21.2) | <0.001 | 13.9 (5.4-35.9) | <0.001 |
| **Annual Income, %** |  |  |  |  |  |  |  |
| <$20,000 | 24% | 115 | 9% | 1 (ref) |  | 1 (ref) |  |
| $20,000-<$75,000 | 56% | 480 | 37% | 1.8 (1.1-2.9) | 0.021 | 0.9 (0.5-1.9) | 0.823 |
| $75,000-<$100,000 | 8% | 225 | 17% | 5.8 (2.9-11.7) | <0.001 | 2.8 (1.0-8.3) | 0.058 |
| ≥$100,000 | 12% | 477 | 37% | 8.2 (4.3-15.9) | <0.001 | 2.9 (1.0-8.6) | 0.057 |
| **Medical conditions, %** |  |  |  |  |  |  |  |
| Hypertension | 73% | 893 | 69% | 0.8 (0.6-1.2) | 0.243 | 1.2 (0.8-1.9) | 0.393 |
| Hyperlipidemia | 71% | 1040 | 80% | 1.6 (1.0-2.8) | 0.06 | 1.8 (0.8-4.3) | 0.143 |
| Diabetes | 30% | 229 | 18% | 0.5 (0.4-0.7) | <0.001 | 0.5 (0.3-1.0) | 0.036 |
| Stroke | 16% | 120 | 9% | 0.6 (0.3-0.9) | 0.021 | 0.7 (0.4-1.2) | 0.207 |
| Heart failure | 25% | 189 | 15% | 0.5 (0.3-0.8) | 0.006 | 0.8 (0.5-1.4) | 0.471 |
| Heart attack | 42% | 638 | 49% | 1.3 (1.0-1.8) | 0.029 | 1.5 (1.0-2.3) | 0.066 |
| **General health, %** |  |  |  |  |  |  |  |
| Excellent | 3% | 77 | 6% | 1 (ref) |  | 1 (ref) |  |
| Very good | 14% | 283 | 22% | 0.8 (0.2-2.6) | 0.68 | 1.0 (0.2-4.2) | 0.945 |
| Good | 43% | 527 | 41% | 0.5 (0.2-1.2) | 0.102 | 0.6 (0.2-2.1) | 0.386 |
| Fair | 29% | 311 | 24% | 0.4 (0.2-1.1) | 0.065 | 0.8 (0.3-2.7) | 0.746 |
| Poor | 12% | 99 | 8% | 0.3 (0.1-0.8) | 0.018 | 0.7 (0.2-3.6) | 0.695 |
| **Smoking, %** |  |  |  |  |  |  |  |
| Never | 39% | 622 | 48% | 1 (ref) |  | 1 (ref) |  |
| Past | 38% | 590 | 45% | 1.0 (0.7-1.4) | 0.902 | 1.7 (0.9-3.0) | 0.084 |
| Current | 23% | 85 | 7% | 0.2 (0.1-0.4) | <0.001 | 0.2 (0.1-0.5) | 0.002 |
| **Sleep duration, h/night** |  |  |  |  |  |  |  |
| ≥9 | 12% | 87 | 7% | 1 (ref) |  | 1 (ref) |  |
| 7 to 8 | 56% | 682 | 53% | 1.7 (1.1-2.5) | 0.013 | 1.2 (0.6-2.6) | 0.57 |
| ≤6 | 32% | 528 | 41% | 2.3 (1.4-3.6) | 0.002 | 2.2 (1.0-4.8) | 0.055 |
| NHANES: National Health And Nutrition Examination Survey; Heh: Health eHeart Study; OR: odds ratio; 95% CI: 95% confidence interval. | | | | | | | |
